# 8p23 beta-defensin copy number determination by single-locus pseudogene-based paralog ratio tests risk bias due to low-frequency sequence variations

**DOI:** 10.1186/1471-2164-15-64

**Published:** 2014-01-24

**Authors:** Xianghong Zhang, Sebastian Müller, Michael Möller, Klaus Huse, Stefan Taudien, Malte Book, Frank Stuber, Matthias Platzer, Marco Groth

**Affiliations:** 1 University Department of Anaesthesiology and Pain Medicine, Bern University Hospital, Inselspital, Bern, Switzerland; 2 Genome Analysis, Leibniz Institute for Age Research – Fritz Lipmann Institute, Jena, Germany; 3 Graduate School for Cellular and Biomedical Sciences, University of Bern, Bern, Switzerland; 4 Systems Biology/Bioinformatics Group, Leibniz Institute for Natural Product Research and Infection Biology - Hans Knoell Institute, Jena, Germany

**Keywords:** Beta-defensin, Copy number variation, Quantitative real-time PCR, Paralog ratio tests, Multiplex ligation-dependent probe amplification, Clustering, Pseudogene, Low frequency sequence variations

## Abstract

**Background:**

The copy number variation (CNV) in beta-defensin genes (DEFB) on human chromosome 8p23 has been proposed to contribute to the phenotypic differences in inflammatory diseases. However, determination of exact DEFB CN is a major challenge in association studies. Quantitative real-time PCR (qPCR), paralog ratio tests (PRT) and multiplex ligation-dependent probe amplification (MLPA) have been extensively used to determine DEFB CN in different laboratories, but inter-method inconsistencies were observed frequently. In this study we asked which one is superior among the three methods for DEFB CN determination.

**Results:**

We developed a clustering approach for MLPA and PRT to statistically correlate data from a single experiment. Then we compared qPCR, a newly designed PRT and MLPA for DEFB CN determination in 285 DNA samples. We found MLPA had the best convergence and clustering results of the raw data and the highest call rate. In addition, the concordance rates between MLPA or PRT and qPCR (32.12% and 37.99%, respectively) were unacceptably low with underestimated CN by qPCR. Concordance rate between MLPA and PRT (90.52%) was high but PRT systematically underestimated CN by one in a subset of samples. In these samples a sequence variant which caused complete PCR dropout of the respective DEFB cluster copies was found in one primer binding site of one of the targeted paralogous pseudogenes.

**Conclusion:**

MLPA is superior to PRT and even more to qPCR for DEFB CN determination. Although the applied PRT provides in most cases reliable results, such a test is particularly sensitive to low-frequency sequence variations preferably accumulating in loci like pseudogenes which are most likely not under selective pressure. In the light of the superior performance of multiplex assays, the drawbacks of such single PRTs could be overcome by combining more test markers.

## Background

Copy number variation (CNV) is very common in human genome [1,2]. Among the many genes affected by CNV, the beta-defensin genes (DEFBs) which are located in chromosome 8p23.1 have been well characterized to be extensively variable in populations [3-6]. These DEFBs form a cluster, and 2 to 12 copies of DEFB cluster were reported in a diploid genome [4,7]. There is a correlation between copy number (CN) and *DEFB4* expression at mRNA level in a variety of cells [4,8-10] i.e. CN are prone to shape phenotypes.

Beta-defensins are a group of cationic antimicrobial peptides. They are mainly expressed in skin and mucus and can be strongly induced after infection, so beta-defensins are considered to contribute to the first line of defense against invading pathogens [11]. In addition, beta-defensins are able to modulate the immune response [12].

Given the functions of beta-defensins in the immune system, researchers have investigated the association between DEFB CN and inflammatory diseases. Increased DEFB CN was reported to be associated with the risk of psoriasis [13,14]. Fellerman *et al.* found an association between low DEFB CN and Crohn’s disease of colon [8]. On the contrary, Bentley et al. reported an association between high DEFB CN and Crohn’s disease [15]. However, none of the associations can be replicated in a recent study by Aldhous et al. [16] which is in main part due to uncertainty in CN determination. The discrepancies raised the necessity to find reliable methods for DEFB CN determination.

So far, it is still a challenge to determine the exact DEFB CN. Three PCR based methods, real-time PCR (qPCR) [8,15,17,18], paralog ratio tests (PRT) [14,16,19-21] and multiplex ligation-dependent probe amplification (MLPA) [7,20,22,23] have being extensively used to determine DEFB CN. qPCR was advantageous due to universal applicability and relative simplicity, but the reliability of this method was questioned [16,22,24]. PRT was designed to avoid the PCR heterogeneity between reference and target genes by using one pair of primers targeting paralogs of a pseudogene [20]. Combined with simplicity and high throughput, PRT is becoming more and more popular, and different designs were used [16,20,24,25]. MLPA has the capacity to interrogate a great deal of locus including target and reference in a single reaction [7,26]. Nevertheless, it is relatively expensive and time-consuming and requires larger amounts of sample DNA. When the CNs determined by these three methods were compared directly in the same samples, inter-method inconsistencies were observed frequently [7,22,24]. Therefore, in this study we asked which one is superior among the three methods for DEFB CN determination. To answer this question, we compared qPCR, PRT and MLPA for DEFB CN determination in 285 DNA samples. Furthermore we investigated the reason for inconsistent CN determination by PRT compared to by MLPA.

## Results

### Integer CN assignment and call rate

**Figure 1:**
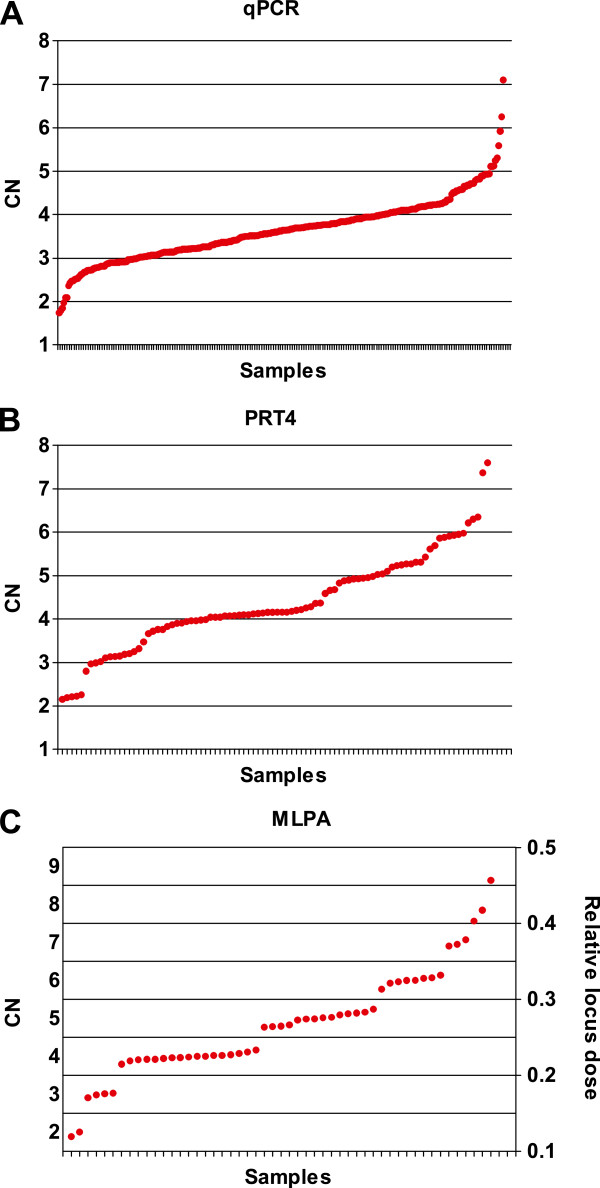
**Scatter plots of raw data of the three methods.** Raw data were plotted in ascending order. **(A)** qPCR - raw CNs of all samples were included. **(B)** PRT4 - raw CNs of samples in a typical 96-well plate were included. **(C)** MLPA - the relative locus dose (right axis) of samples in a typical batch were included.

In order to compare the performance of qPCR, PRT4 and MLPA, DEFB CNs were determined in DNA from blood of 285 healthy Europeans (see Additional file 1). To evaluate the distribution of the raw data of each method individually, the raw CNs of all samples from qPCR (with the calibrator included in each run), the raw CNs of a typical 96-well plate from PRT4 and the relative locus dose of a typical batch from MLPA were plotted in an ascending order (Figure 1). The distributions of the raw CNs of the other PRT4 plates and the relative locus dose of the other MLPA batches are shown in Additional file 2 and Additional file 3, respectively. In qPCR, the raw CNs are distributed continuously, and no clustering around integer CNs was observed. In contrast, for MLPA the relative locus dose increases stepwise and clear intervals between neighboring CN clusters are visible as shown in the plot as “gaps”. For PRT4, a plot between these two extremes was obtained.

**Figure 2:**
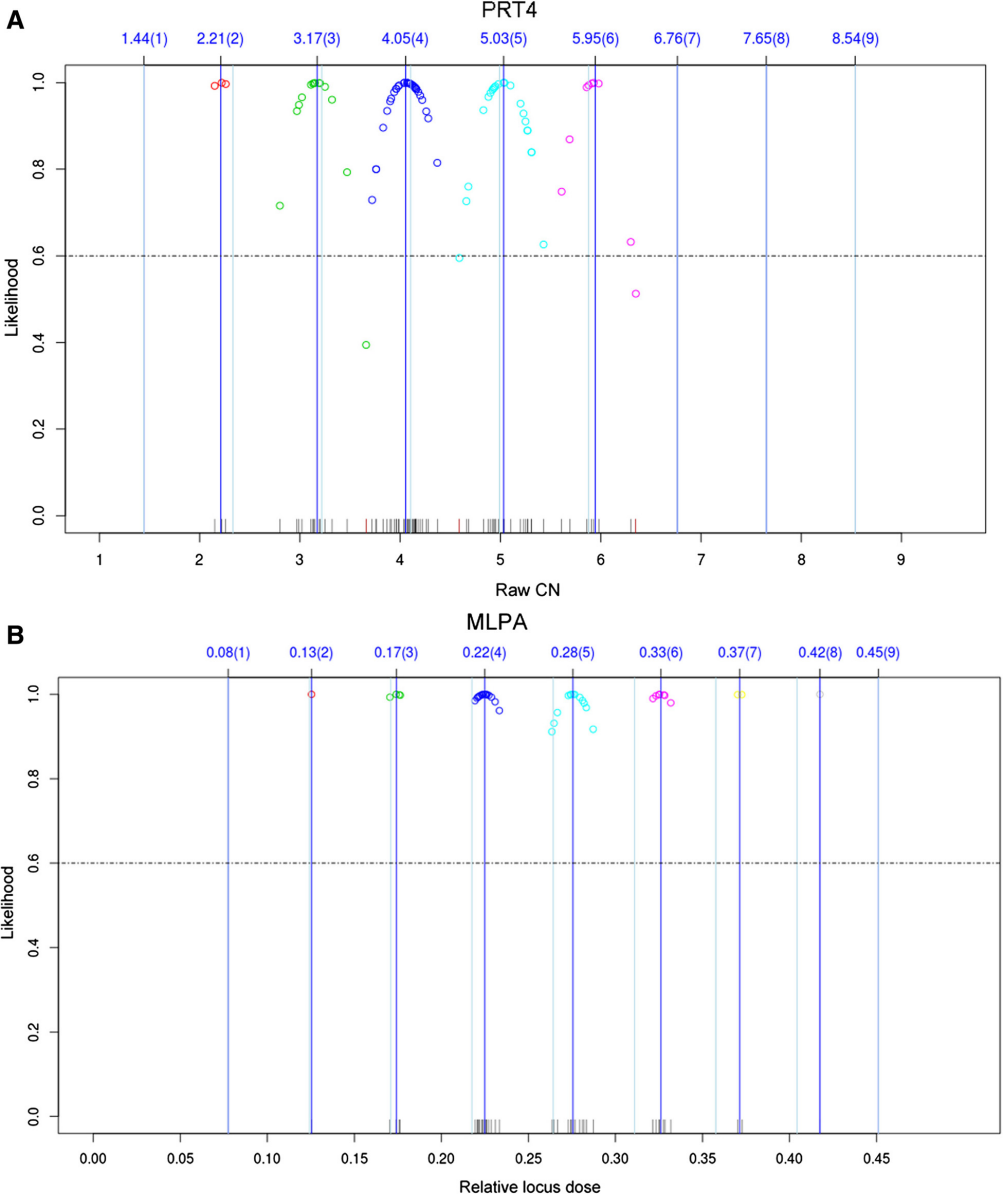
**Integer CN assignment using a clustering algorithm for (A) PRT4 and (B) MLPA.** Results of a typical 96-well plate/batch are shown for PRT4 and MLPA, respectively. The dark blue lines indicate the means of the clusters with their values above and assigned integer CNs in brackets. The light blue lines indicate the primary cluster center obtained from reference samples. Circles with the same color represent one cluster. The dashed line indicates the threshold of likelihood below which samples were discarded.

**Figure 3:**
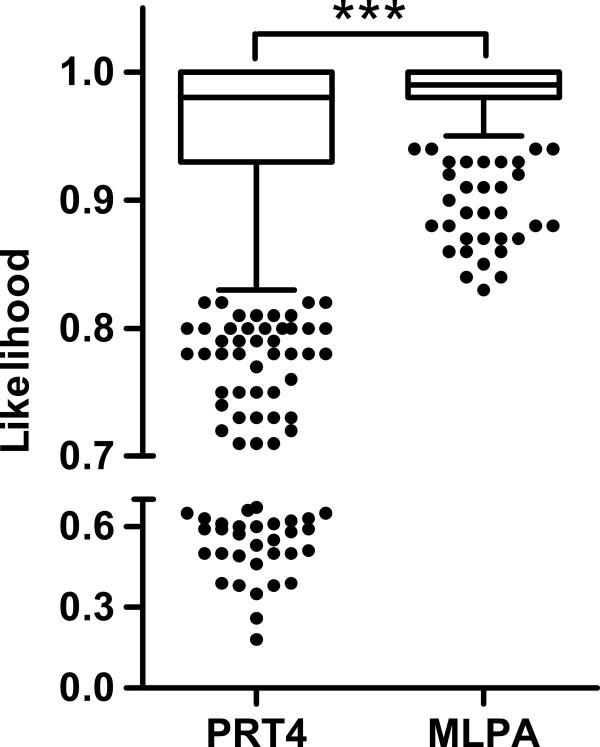
**Likelihood values of PRT4 and MLPA shown as box plots.** Box represents the region between the first and the third quartile (i.e. the central 50 % of the data). The horizontal line in the box represents the median. The end of the vertical line marks the smallest likelihood value located less than 1.5 times the interquartile distance from the first quartile. Other observations may be deemed outliers and are shown as dots. ***P < 0.001 in a Mann–Whitney test.

For qPCR, raw CN were rounded to the nearest integer. For PRT4 and MLPA, a clustering algorithm was applied creating likelihood values for integer assignments. In Figure 2, the clustering results of a typical 96-well plate/batch were shown for PRT4 and MLPA, respectively. The distribution of raw data around the cluster centers is broader for PRT4 than for MLPA. Accordingly, the likelihood value of PRT4 was significantly lower than that of MLPA (Figure 3). Moreover, 9.8% of the samples did not pass the threshold of likelihood in PRT4. In contrast, all but two samples passed in MLPA.

The qPCR and PRT4 assays were performed twice and samples were considered if the results from the duplicates were consistent. The call rate of MLPA was with 99.3% the highest among three methods in contrast to qPCR (96.8%) and PRT4 (82.1%). Among uncalled samples in PRT4, 78.4% showed an MPLA-CN ≥ 5.

### Inter-method concordance and discordance

**Figure 4:**
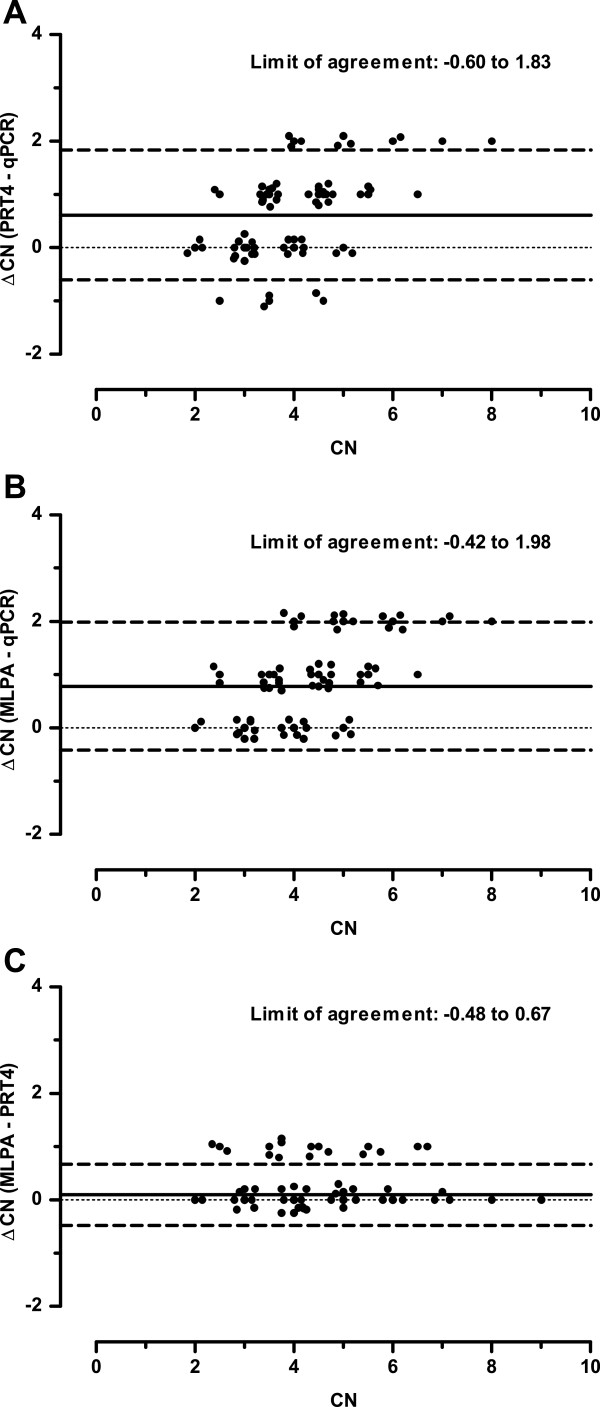
**Bland-Altman plots for inter-method comparisons of (A) PRT4-qPCR (B) MLPA-qPCR and (C) MLPA-PRT4.** The plots show the difference between the methods against their mean. The dots were offset to allow individual measurements to be distinguished. The solid line represents the mean of all ΔCNs and the dashed line is 1.96 times the standard deviation (limit of agreement).

Concordance and discordance between methods were evaluated by pairwise comparison of CNs (Table 1). For visualization of the inter-method difference, Bland-Altman plots were applied (Figure 4). The concordance rates among three method pairs were significantly different (*P* < 0.0001; Chi-square test). The concordance rate between MLPA and PRT4 (90.5%) was the highest. Accordingly, the range of limit of agreement between MLPA and PRT4 (-0.48 to 0.67) is the smallest. In comparison to MLPA, qPCR underestimated the CN (on average by 0.78), with greater underestimation at higher CN. When PRT4 and qPCR CNs were compared, we observed a very similar trend.

**Table 1:** Concordant and discordant results between methods

**ΔCN**	**-1**	**0**	**+1**	**+2**
PRT4 - qPCR	6 (2.6%)	**87 (38.0%)**	125 (54.6%)	11 (4.8%)
MLPA - qPCR	0	**88 (32.1%)**	158 (57.7%)	28 (10.2%)
MLPA - PRT4	0	**210 (90.5%)**	22 (9.5%)	0

**Figure 5:**
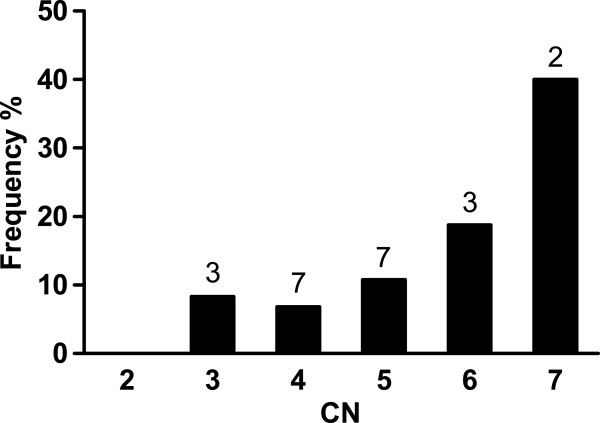
**Frequency of samples underestimated by 1 copy by PRT4 in different CN groups.** MLPA CNs were regarded as true. Absolute numbers are shown above the columns.

Comparing MLPA and PRT4, in all 22 discordant cases (9.5%) the PRT underestimated the CN by 1. In addition, a linear trend between CN and the fraction of underestimated samples is very likely (*P* = 0.023, Chi-square test for trend, Figure 5).

### Genomic variation in a PRT4 primer binding site

**Figure 6:**
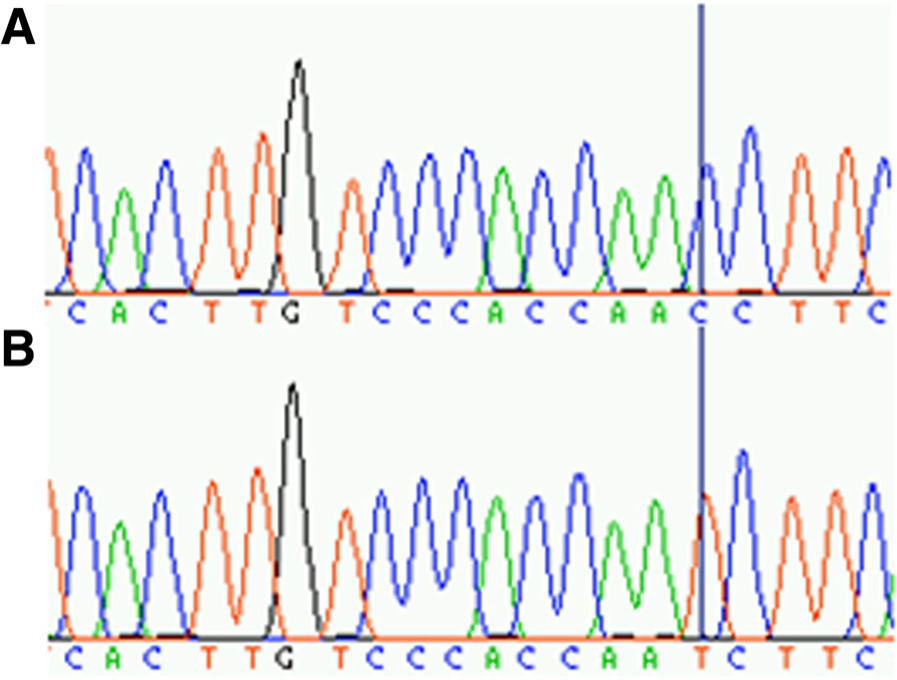
**Paralogous sequence variation C > T (PSV, chr8: 7277574, rs187261177) in the PRT4 forward primer binding site (5’-CACTTGTCCCACCAACCTTC).** Sequencing traces of two clones derived from sample RC081 (CN = 5 by MLPA, CN = 4 by PRT4) represent **(A)** the major and **(B)** the minor PSV allele.

**Figure 7:**
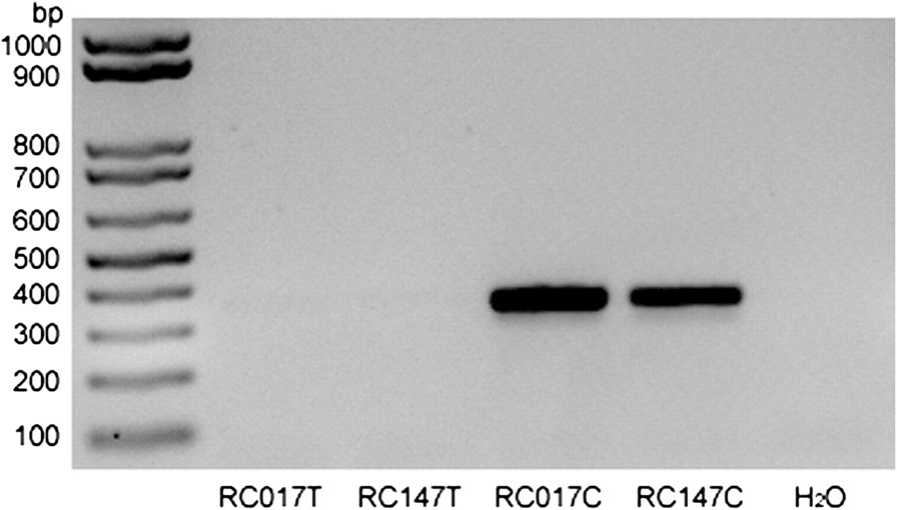
**PCR products from 4 clones under PRT4 conditions.** RC017T and RC147T indicate 2 clones containing T allele amplicons from RC017 and RC147. RC017C and RC147C indicate 2 clones containing C allele amplicons from RC017 and RC147. H_2_O means a non-template control.

As the discordance between MLPA and PRT4 could be confirmed for selected samples repeatedly, we speculated that PRT4 may systematically miss a particular target allele in the DEFB region. Therefore, 10 samples discordant between MLPA and PRT4 were selected for variation screening in the PRT4 primer binding sites by sequencing. A C > T paralogous sequence variation (PSV, chr8: 7277574, rs187261177) was found in the forward primer binding site in 9 samples (Figure 6, Table 2). The variant was not found among 65 clones obtained from the genomic DNA pool which indicates a significant difference in the rs187261177 minor allele frequency (MAF) among random samples and those 10 selected for their MLPA/PRT4 discordance (*P* < 0.001, Fisher’s exact test). To test whether the T allele was missed in PRT4, 2 clones containing T allele amplicons from RC017 and RC147 and 2 clones containing C allele amplicons from RC017 and RC147 identified by sequencing were used as test samples and control samples, respectively. The result indicates that the T allele cannot be amplified by the PRT4 PCR (Figure 7), even in the artificial condition of an extremely low complexity and high-copy template.

**Table 2:** 10 samples and genomic DNA pool for variation screening in primer binding site in PRT4

**Sample**	**MLPA CN**	**PRT4 CN**	**Clones sequenced**	**Clones with variation**
RC017	3	2	10	3
RC147	3	2	11	5
RC191	4	3	11	2
RC206	4	3	12	4
RC081	5	4	26	6
RC287	5	4	31	5
RC006	6	5	41	5
RC020	6	5	36	4
RC011	7	6	41	0^1^
RC121	7	6	84	2
**Sum**	**50**	**40**	**303**	**36**
Pool	n.d.	n.d.	65	0

^1^Number of sequenced clones too low for variation detection with high power. n.d. means not determined.

### CN distributions from three methods

**Figure 8:**
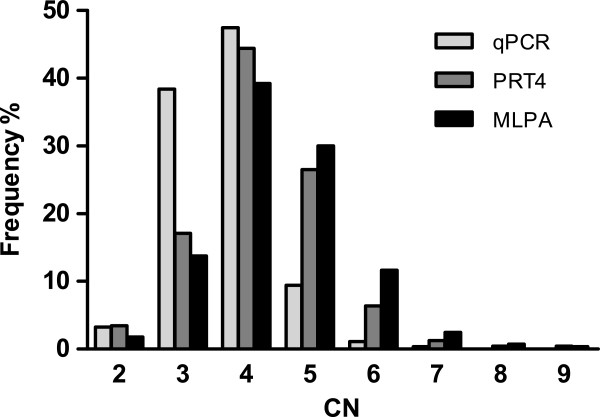
DEFB CN distributions from three methods.

Compared to the CN distribution obtained by MLPA, the qPCR and PRT4 patterns are shifted towards low CNs (Figure 8). Although the medians from the three methods were all 4, they were statistically different (*P* < 0.0001; Kruskal-Wallis test). Multiple comparisons qPCR *vs* PRT4, qPCR *vs* MLPA and PRT4 *vs* MLPA also showed significant differences (*P* < 0.001, *P* < 0.001 and *P* < 0.05, respectively; post-hoc Dunn’s test).

## Discussion

In the present study, we compared the call rates and concordance of qPCR, PRT4 and MLPA for the DEFB CN determination. We herewith extended a previous study [22] where qPCR and MLPA were explored on 42 cell line and 38 blood derived samples. In the present study, we included a PRT as respective approaches which are combining simplicity and high throughput and becoming popular in the field [16,20,24]. Moreover, we extended the test sample considerably to 285 DNAs isolated from blood of healthy donors with European ancestry. Furthermore, a clustering algorithm was used to infer the integer CN from raw data in PRT4 and MLPA with a threshold of confidence.

In respect to raw data, we found the best convergence for MLPA. “Gaps” of locus dose between neighboring clusters provide discriminatory power to the method resulting in higher confidence for the CN estimation compared to PRT4. Nearly no clustering of raw data at all was observed for qPCR, which confirmed the findings of previous reports [16,22,24]. It results in frequent typing errors and serious shortcomings of this method. qPCR systematically underestimated in particular higher CNs, which confirmed our results in a previous report [22].

For this study, we developed a clustering algorithm with confidence of determination to infer CN from raw data of PRT4 and MLPA. To infer CN from raw data, rounding to the nearest integer [5,17], linear regression by integrating reference samples [18,20,24] and Gaussian mixture modeling [16] were used. Signal saturation as the CN increases was reported in PPRT and MLPA [7]. So directly rounding the raw data to the nearest integer may underestimate the CN, and linear regression may overestimate or underestimate the CN. Gaussian mixture modeling was well established for CN calling from large data sets. For small data sets, however, CN calls will be biased particularly for high CNs due to the low data amount in the clusters to form Gaussian curves. In extreme cases of no data in one or even more clusters, the Gaussian curves for adjacent clusters will be remarkably affected and CNs will be misestimated eventually. In the PRT4 and MLPA assays, to completely avoid variations between plates or batches, analysis was performed in a plate- or batch- wise manner, so the data sets were too small to call CNs using the Gaussian mixture model. In addition, due to differences between primary cluster centers determined from reference samples and actual cluster centers determined from the test samples, CNs called by Gaussian mixture modeling using reference samples will also be misestimated. In contrast, these misestimations can be avoided by the developed clustering approach, which furthermore allows setting a threshold of confidence enhancing the reliability of the determination.

MLPA had the highest call rate of > 99%, which is higher than that reported in triplex PRT (95%) in which also a likelihood analysis with a threshold of confidence was applied [16]. In contrast, the PRT4 applied by us had a considerably lower call rate (only 82%) especially for high CNs. It was considered that loss of large amount of data or/and biased loss of data between cases and controls could generate spurious associations in CNV association studies [16]. In case of qPCR, the initial low call rate of 84% could be increased by a second run to almost 97%. Possibly, the call rate of PRT4 could also be increased by repeating the assay several times, but in contrast to qPCR it is time and labor consuming.

Accordingly, we found a high concordance rate between MLPA and PRT4 but a lower one between MLPA or PRT4 and qPCR. Most of the MLPA and PRT4 CNs agreed except 22 cases where PRT4 missed exactly one DEFB copy. We could explain this phenomenon by identifying a PSV in the PRT4 forward primer which completely abolished amplification from DEFB clusters containing the minor T allele. Unknown during the assay design and the course of the work it turned out to be identified recently by the 1000 genomes project as rs187261177, although no minor allele frequency was reported yet. Missing this PSV among 65 clones from the DNA pool of 80 individual DNA indicates that it is a low-frequency variation. In respect to the 232 samples with both MLPA CNs and PRT4 CNs in the present study, comprising altogether 1003 copies of the DEFB cluster, the minor PSV allele frequency is 2.2%. Assuming a random distribution of this PSV allele among DEFB CN alleles (e.g. chromosomes with a particular copy of the DEFB cluster) would result in linearly increased frequency of samples underestimated with copy numbers, e.g. the probability to underestimate a CN by 1 is four times higher for a 8-copy genome compared to a 2-copy genome. This assumption is supported by the linear trend between CN and the frequency of samples underestimated we observed (Figure 5) although the sample size (232 individuals) is small.

Most remarkably, the CN distributions of the three methods differed significantly. Among them, the distribution from MLPA is very comparable to the recently reported distribution obtained by triplex PRT in European populations [14,24]. The distribution from qPCR shifted to low CNs due to underestimation of CNs, whereas a similar shift for PRT4 is caused by its low call rate at high CNs and underestimation by one copy in the samples carrying the PSV rs187261177 at the primer binding site.

There are several advantages of MLPA over PRT and/or qPCR in design. The first is given by the usage of several markers. Ten markers within the DEFB cluster and 25 single-copy reference loci were used to interrogate the CN in MLPA in contrast to only one marker in qPCR and PRT4. Different behaviors of different markers inducing discrepant CN estimations were reported for qPCR [22] and PRT [16]. Therefore, in our opinion the combination of numerous markers is a crucial prerequisite to achieve a comprehensive and accurate estimation of CN by avoiding bias(es) from single markers. This assumption is supported by the CN determination with triplex PRT [16,24]. In this method, three markers were included and raw data showed good convergence around the cluster centers. Moreover, CNs from triplex PRT were consistent with those from MLPA in a panel of DNA samples (see Table 1 in FODE *et al.* 2011). Based on this assumption, the PRT4 assay could be combined with other assays, e.g. previously established PRTs [16,20] to avoid the bias from the PSV rs187261177 in primer binding site and further improve the performance for CN determination.

Another advantage of MLPA is evident from the described PRT4 CN determination problem due to a PSV at the primer binding site. MLPA probe targeting sites are usually located in functional genes which are under strong negative selection and whose variations are well characterized. Furthermore, it was shown that variations at the probe hybridization site only influence its efficiency if positions directly adjacent to the ligation site are affected [26]. However, in PRT4, the pseudogene *HSPDP3* is the target and a PSV in the primer binding site in one copy of the DEFB clusters resulted in complete PCR dropout of respective copy and consequent underestimation of CN of samples. Pseudogenes, especially retrotransposed pseudogenes lacking active promoters, represent putatively unconstrained sequences which are accumulating variations due to the lack of selection pressure [27,28]. SNP density and *K*_a_/*K*_s_ ratios of pseudogenes are significantly higher than those of genes [29] and, overall, variation in pseudogenes is less well characterized because they are not common targets of resequencing projects as genes are. Accordingly, it is likely that unknown variations may compromise primer binding sites. Furthermore, processes like gene conversion and/or non-allelic homologous recombination between different pseudogenes of the same family (and with the functional gene) may further increase the genetic variability within primer binding sites. In addition, the high annealing temperatures applied to ensure the PCR specificity make the PRT assays susceptible to the sequence variations in primer binding sites. The problem in PRT4 is also seen with the PRT applied for the estimation of DEFB CN [20]. We identified rs56784821 as being present on one DEFB copy in the HapMap/CEPH sample NA18502 causing underestimation of its CN by one (data not shown) [7]. In addition, the PSV rs187261177 interfering with our PRT4 assay is also located within the reverse primer binding site of HSPD21-PRT [16] although at the 5’ end. Therefore, it is possible that the low call rate in PRT4 is due to additional primer binding site variants in the target locus and/or its paralogs on other chromosomes. Altogether, these results suggest that pseudogenes should be avoided as targets for paralog ratio tests whenever possible.

## Conclusions

Although expensive and time consuming, MLPA is superior to qPCR and PRT4 for DEFB CN determination. If accuracy has the highest priority, it is the best method to be applied in association studies and the raw data should be subjected to cluster analysis using thresholds of confidence.

## Methods

### Genomic DNA

A number of 285 healthy blood donors of European ancestry were enrolled in this study. This study was approved by the ethics committee of the Canton Bern, Switzerland. Written informed consent from all of the participants was obtained. Genomic DNA was isolated from peripheral whole blood using QIAamp DNA-blood Mini Kit (Qiagen, Hilden, Germany) according to manufacturer’s instructions. A human genomic DNA pool (Roche, Mannheim, Germany) containing DNA from 80 healthy individuals was also purchased. In addition, four DNAs (NA18552, NA15324, NA12760 and NA18858) with known CN (2, 4, 6 and 8, respectively) were isolated from commercially available lymphoblastoid cell lines (Coriell Cell repository http://www.coriell.org/). Reliable copy number details of these samples are from independent, methodologically different determinations from different laboratories (see Table 2 in Groth *et al.* 2008 and references therein). The concentration and purity of DNA were determined by a NanoDrop spectrophotometer (Thermo Scientific, Wilmington, USA). A260/A280 ≥ 1.7 and A260/A230 ≥ 1.5 were reached in all samples. The concentration ranged from 50 to 200 ng/μl.

### qPCR

qPCR was modified from our previously established method by targeting *DEFB4*[17]. In brief, the target locus in *DEFB4* and the reference locus in the human albumin gene (*ALB*) were amplified simultaneously in a duplex PCR. The primers and probes as well as the PCR conditions were the same as we used in a previously established method [17]. PCR was performed in duplication for each sample. Genomic DNAs NA18552 and NA15324 were used as calibrator and positive control, respectively. The PCR efficiencies for target and reference genes were determined by amplifying a randomly selected sample in 10-step dilution series. The calibrator and positive control were included in each PCR run. Gene dosage ratio between *DEFB4* and *ALB* can be determined by integrating Cp values (cycle number when the signal reaches the threshold) and PCR efficiencies for two genes. Raw CN was determined by normalizing the gene dosage ratio of unknown samples to that of the calibrator. This calculation was performed by the Lightcycler relative quantification software 1.0 (Roche). Raw CN were rounded to the nearest integer. Only when the positive control showed the correct CN, the CNs of unknown samples were deemed to be correct. In addition, only when the duplicated PCRs for unknown sample showed consistent CNs, the result was accepted. A second run with PCR in duplication was performed for the samples with inconsistent CNs in the first run.

### PRT

**Figure 9:**
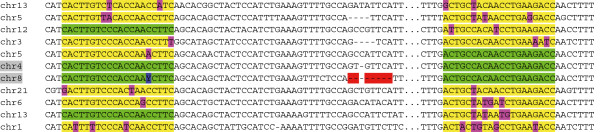
**PRT4 primer design and locus specificity.** Partial sequences of HSPDP3 paralogs on different chromosomes are shown. The primers were designed to amplify loci only on chromosome 8 (target) and chromosome 4 (reference). Gray: amplified loci; green: primer binding site with perfect match; yellow: primer binding site with mismatches; purple: mismatch; red: deletion which generates the length difference of PCR products; blue: paralogous sequence variation rs187261177.

Our PRT (termed as PRT4) was designed on the basis of the PRT described by Armour *et al.*[20] targeting *HSPDP3*. The paralog in the DEFB cluster on chromosome 8 (target locus) and the paralog on chromosome 4 (reference locus) were exclusively amplified by using specific primers (Figure 9): 5’-CACTTGTCCCACCAACCTTC-3’ (forward) and 5’-GGTCTTCAGGTTGTGGCAGT-3’ (reverse). The reverse primer was 5’-6-carboxyfluorescein (FAM)-labeled. PCR was performed as follows: pre-denaturation at 95°C for 3 minutes, 26 cycles of denaturation at 93°C for 30 seconds followed by annealing and elongation at 68°C for 1 minute, final elongation at 72°C for 45 minutes and cooling at 18°C for 2 minutes. Two independent PCRs separated in two 96-well plates were performed for each sample. Genomic DNAs NA18552, NA15324, NA12760 and NA18858 were included as reference samples in all plates. The PCR products from target and reference locus can be distinguished by length, so they can be analyzed in capillary electrophoresis. The FAM-labeled PCR products were appropriately diluted (up to 1/40) and 1 μl of the dilution was supplemented with 10 μl formamide (Roth, Karlsruhe, Germany) and 0.5 μl of GeneScan ROX 500 marker (Applied Biosystems, Darmstadt, Germany). The mixture was incubated at 94°C for 3 min, and the denatured PCR products were then separated on an ABI 3730 capillary sequencer and analyzed with the GeneMapper 4.0 software (Applied Biosystems). The amount of each amplicon within a PCR reaction was calculated by the respective area under the curve. Subsequent calculations of raw CNs are the ratios of areas from target locus amplicon and reference locus amplicon assuming a CN of two for the reference locus (CN = 2 × target/reference).

### MLPA

MLPA was performed as described [7] using the SALSA MLPA P139-B1 Defensin kit (MRC Holland, Amsterdam, The Netherlands). The MLPA probe set consists of 40 probes of which 5 are hybridizing to genes/pseudogenes within alpha-defensin (DEFA) cluster, 10 to genes within DEFB cluster and 25 to *bona fide* single-copy genes on chromosome 8 as well as on other chromosomes, respectively. In brief, hybridization, ligation, amplification and electrophoresis were carried out. Peak areas were normalized against the summed peak areas of the “five nearest neighbor” (5nn) reference probes to obtain 5nn values for each individual sample, and relative locus doses of DEFB cluster were calculated by averaging the 5nn values of the 10 probes targeting DEFB cluster. Genomic DNAs NA18552, NA15324, NA12760 and NA18858 were included as reference samples in all experiements/batches. DEFB CNs were determined in a batch-wise manner. Each batch included reference samples as described and a number of 30–50 unknown samples. One measurement was performed for each test sample.

### Cluster analysis

To infer integer CN from experimental values (raw CNs of PRT4 and relative locus doses of MLPA), a cluster algorithm was developed. The details of this algorithm are introduced as follows. Firstly, the values of the reference samples with even CNs were determined experimentally. The values of reference samples with odd CNs were interpolated by a linear regression. Those reference values were used as primary cluster means. Then the values of unknown samples in each 96-well plate (PRT4) or batch (MLPA) were clustered to the closest primary cluster means. The new cluster means were calculated after clustering. Furthermore, the second closest cluster mean for each value was identified to determine the likelihood for its assignment. This likelihood is expressed as follows: square of the distance to the second closest cluster mean/(square of the distance to the closest cluster mean + square of the distance to the second closest cluster mean). This formula implies that the more similar the distances of a value to its closest and second closest cluster mean are, the lower is the likelihood to determine the true value. The minimal threshold of the likelihood was set to 0.6 for both PRT4 and MLPA. In PRT4, only when the CNs from two independent PCRs were consistent, the CN was deemed to be reliable. This clustering algorithm was run under R (http://www.r-project.org/) on a UNIX platform. The program can be downloaded from http://genome.fli-leibniz.de/software.

### Mutation screening in primer binding site in PRT4

To inspect primer binding sites in the DNA pool (Roche) and DNA samples with CNs consistently differing by one between PRT4 and MLPA, PCR was carried out with primers outside of PRT4 targeted regions: 5’-CAATGCCTTCTTCAACAGCA-3’ (forward) and 5’-AATGTGAATTCCAGGATGCC-3’ (reverse). PCR was performed using 50 ng of template DNA. PCR conditions were as follows: pre-denaturation at 95°C for 1 minute, 30 cycles of denaturation at 95°C for 30 seconds followed by annealing at 59°C for 30 seconds and elongation at 72°C for 1 minute, final elongation at 72°C for 30 minutes and cooling at 18°C for 1 minute. PCR products were precipitated with ammonium acetate and ethanol, dried and re-dissolved in water. For primers amplifying several *HSPDP3* paralogs direct amplicon sequencing was not feasible. Accordingly, the amplicon was cloned into pCR2.1 vector with the TOPO TA Cloning Kit (Invitrogen, Darmstadt, Germany) according to the manufacturer's instructions. Well isolated white colonies were picked and grown in LB Broth supplemented with ampicillin. Plasmid DNA was isolated from the cultures by automated BioRobot 8000 (Qiagen) and inserts were sequenced in both directions using M13 universal primers. PRT4 target amplicons were identified by their sequences and visualized/analyzed using Genome Assembly Program (GAP) [30]. About two-thirds of all clones derived from chromosome 8 and could be evaluated, and the main contaminations are from pseudogene copies from chromosomes 6, 12 and 16.

### Statistical analysis

Statistical analyses and plotting were performed using GraphPad Prism 5.01 for Windows (GraphPad Software, San Diego, CA). The call rates (the samples with determined CNs/all the samples) of three methods were compared using Chi-square test. A comparison of likelihood values in clustering analysis of PRT4 and MLPA was performed using Mann–Whitney test. The inter-method concordance rates (the samples with consistent CNs between two methods/the samples with CNs determined by both methods) were compared using Chi-square test, and the inter-method differences were visually analyzed by Bland-Altman plots. Comparisons of CN from three methods were performed using Kruskal-Wallis test with post-hoc Dunn’s test. All the statistical tests were two-tailed, and *P* values < 0.05 were considered as statistically significant.

### Availability of supporting data

The data sets supporting the results of this article are included within the article and its additional files.

## Competing interests

The authors declare that they have no competing interests.

## Author’s contributions

XZ typed CN of 285 samples by three methods, and analyzed the data and drafted the manuscript. SM developed the clustering program. MM participated in the development of PRT4 assay. KH developed the PRT4 assay and screened the mutation in primer binding site. MB collected the samples. FS and MP participated in the design of the study. MG initiated and coordinated the study. KH, ST, MB, FS, MP and MG revised the manuscript. All authors read and approved the final manuscript.

## Supplementary Material

Additional file 1 **DEFB CN determined by qPCR, PRT4 and MLPA in 285 healthy Europeans.** Raw data for each sample with each method were included. Click here for file

Additional file 2 **Scatter plots of the raw CNs of the other PRT4 plates.** The raw CNs were plotted in ascending order. Click here for file

Additional file 3 **Scatter plots of the relative locus dose of the other MLPA batches.** The relative locus doses were plotted in ascending order. Click here for file
