# Supplementary figures and images for: 8p23 beta-defensin copy number determination by single-locus pseudogene-based paralog ratio tests risk bias due to low-frequency sequence variations
Source: BMC Genomics. 2014 Jan 24;15:64. doi: 10.1186/1471-2164-15-64 (PMC3937106; doi:10.1186/1471-2164-15-64)

**Plate 1-2**

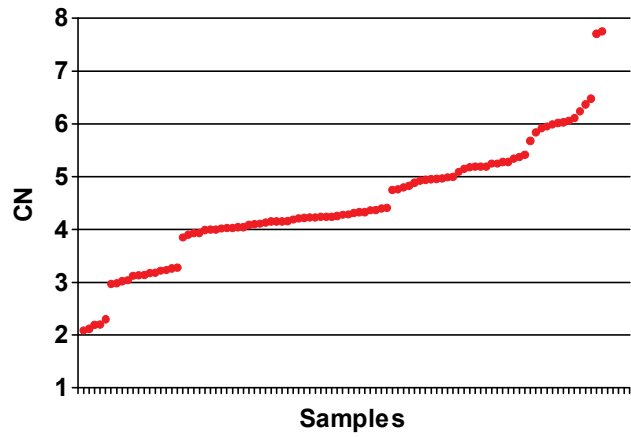

**Plate 2-1**

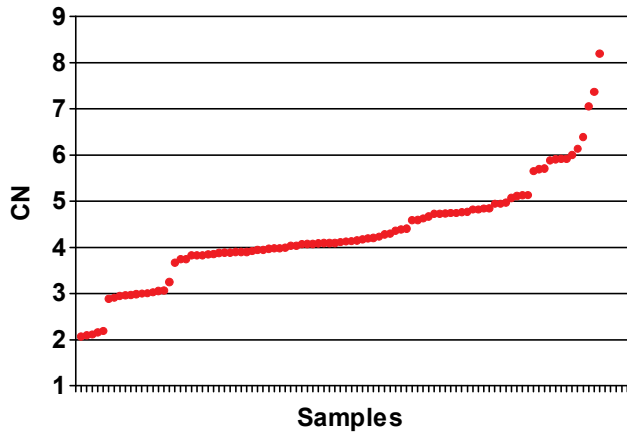

**Plate 2-2**

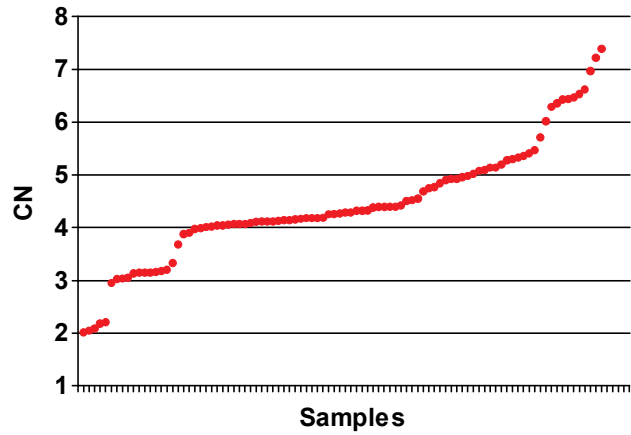

**Plate 3-1**

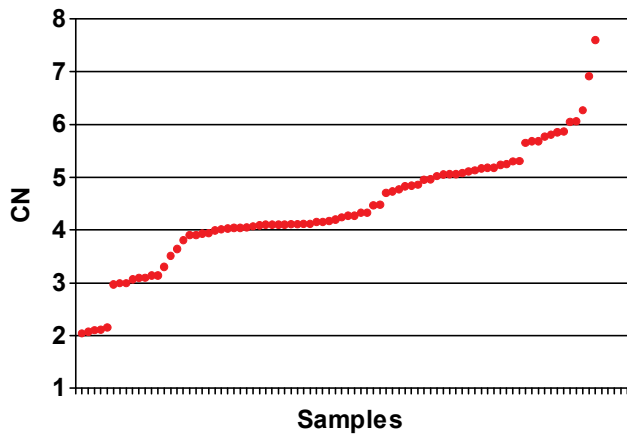

**Plate 3-2**

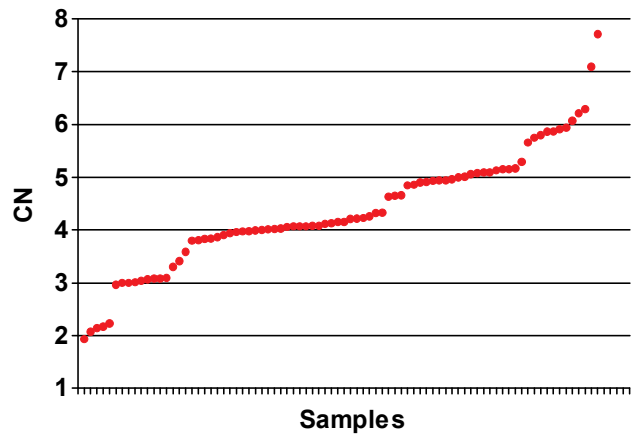

Supplement: Additional file 2 — Scatter plots of the raw CNs of the other PRT4 plates. The raw CNs were plotted in ascending order. [file 1471-2164-15-64-S2.pdf]

Batch 2

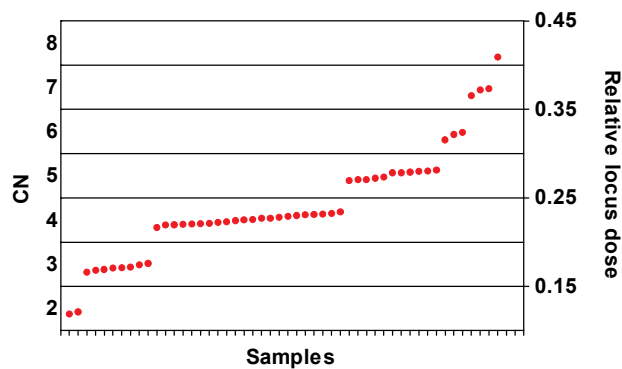

Batch 3

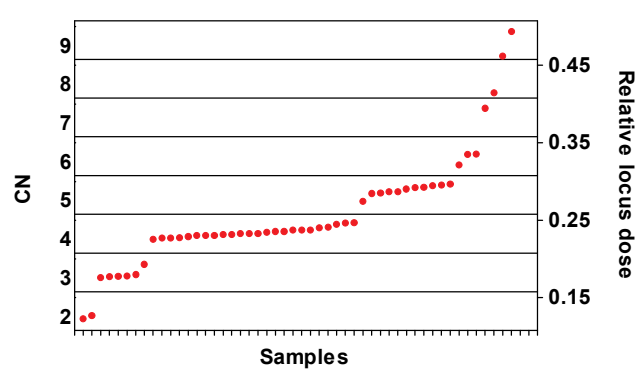

Batch 4

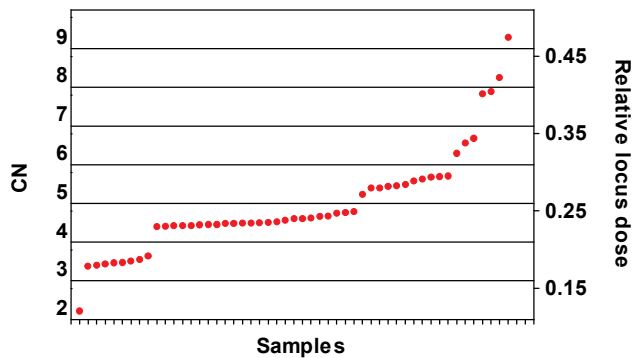

Batch 5

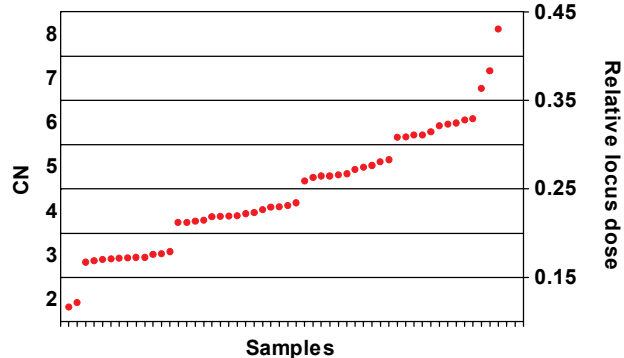

Batch 6

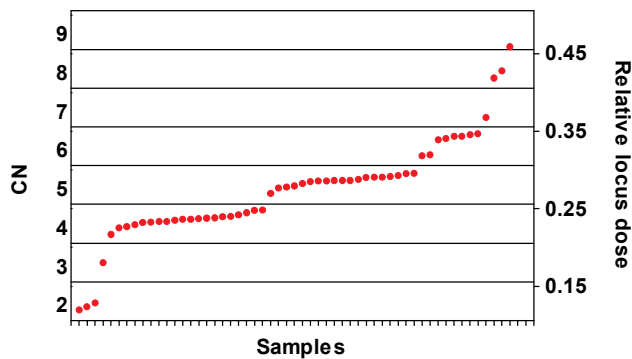

Supplement: Additional file 3 — Scatter plots of the relative locus dose of the other MLPA batches. The relative locus doses were plotted in ascending order. [file 1471-2164-15-64-S3.pdf]
